# Low expression of ELOVL6 may be involved in fat loss in white adipose tissue of cancer-associated cachexia

**DOI:** 10.1186/s12944-024-02126-9

**Published:** 2024-05-17

**Authors:** Chenyang Jin, Shuangjie Wang, Xiangyu Sui, Qingyang Meng, Guohao Wu

**Affiliations:** 1 Department of General Surgery, Zhongshan Hospital of Fudan University, Shanghai, China; 2 Shanghai Institute for Biomedical and Pharmaceutical Technologies, Shanghai, China; 3 Shanghai Clinical Nutrition Research Centre, Shanghai, China

**Keywords:** Cancer-associated cachexia, ELOVL6, Fat loss, Long-chain fatty acid

## Abstract

**Background:**

Cancer-associated cachexia (CAC) arises from malignant tumors and leads to a debilitating wasting syndrome. In the pathophysiology of CAC, the depletion of fat plays an important role. The mechanisms of CAC-induced fat loss include the enhancement of lipolysis, inhibition of lipogenesis, and browning of white adipose tissue (WAT). However, few lipid-metabolic enzymes have been reported to be involved in CAC. This study hypothesized that ELOVL6, a critical enzyme for the elongation of fatty acids, may be involved in fat loss in CAC.

**Methods:**

Transcriptome sequencing technology was used to identify CAC-related genes in the WAT of a CAC rodent model. Then, the expression level of ELOVL6 and the fatty acid composition were analyzed in a large clinical sample. *Elovl6* was knocked down by siRNA in 3T3-L1 mouse preadipocytes to compare with wild-type 3T3-L1 cells treated with tumor cell conditioned medium.

**Results:**

In the WAT of patients with CAC, a significant decrease in the expression of ELOVL6 was found, which was linearly correlated with the extent of body mass reduction. Gas chromatographic analysis revealed an increase in palmitic acid (C16:0) and a decrease in linoleic acid (C18:2n-6) in these tissue samples. After treatment with tumor cell-conditioned medium, 3T3-L1 mouse preadipocytes showed a decrease in Elovl6 expression, and *Elovl6*-knockdown cells exhibited a reduction in preadipocyte differentiation and lipogenesis. Similarly, the knockdown of *Elovl6* in 3T3-L1 cells resulted in a significant increase in palmitic acid (C16:0) and a marked decrease in oleic acid (C18:1n-9) content.

**Conclusion:**

Overall, the expression of ELOVL6 was decreased in the WAT of CAC patients. Decreased expression of ELOVL6 might induce fat loss in CAC patients by potentially altering the fatty acid composition of adipocytes. These findings suggest that ELOVL6 may be used as a valuable biomarker for the early diagnosis of CAC and may hold promise as a target for future therapies.

**Supplementary Information:**

The online version contains supplementary material available at 10.1186/s12944-024-02126-9.

## Introduction

Cancer is one of the most common causes of death worldwide and led to almost 10 million deaths in 2020 [1]. The metastasis and rapid proliferation of tumor cells can cause multiple systemic syndromes, including cancer-associated cachexia (CAC) [1]. CAC is characterized as a wasting syndrome induced by malignant tumors [2–4]. It involves persistent skeletal muscle depletion and fat loss, which are challenging to fully reverse using standard nutritional interventions. CAC not only leads to a grim prognosis but also diminishes the quality of life in individuals with malignant tumors [5, 6]. However, our understanding of the mechanisms underlying CAC remains relatively limited. Previous laboratory and clinical studies have revealed the generation of proinflammatory cytokines during crosstalk among somatic cells, immune cells, and tumor cells. These cytokines may adversely affect skeletal muscle, adipose tissue, and the central nervous system, causing clinical symptoms such as decreased appetite, skeletal muscle atrophy, and fat loss [7]. Recently, the role of fat loss in the pathophysiology of CAC has been reported as an increasingly important issue. Recent studies using CAC rodent models suggest that fat loss and altered lipid metabolic processes can occur before skeletal muscle atrophy [8–12]. White adipose tissue (WAT) is the most important energy storage site in adults and secretes various cytokines and hormones that regulate inflammation and metabolism [13]. According to most studies, the loss of WAT in CAC occurs primarily through three mechanisms: enhancement of fat hydrolysis, inhibition of fat synthesis, and browning of WAT [7, 14].

In this study, transcriptome sequencing of WAT from tumor-bearing mice was carried out. A reduction in the expression level of elongase of very long-chain fatty acid (ELOVL6) was then observed. Previous studies have found that ELOVL6 is abundant in lipid-rich tissues such as WAT and serves as the enzyme responsible for extending saturated fatty acids comprising 12–16 carbon atoms [15–17]. In rodent models, multiple studies have indicated that Elovl6 plays a critical role in regulating fatty acid metabolism. Notably, *Elovl6* knockout mice are protected from diet-induced insulin resistance [18, 19]. Under cold-acclimated conditions, *Elovl6* knockout mice exhibit reduced expression of genes associated with the thermogenic process in brown adipose tissue (BAT), indicating impaired thermogenic capacity [20]. Furthermore, over the past few decades, numerous studies have highlighted the crucial role of long-chain fatty acids (LCFAs) in regulating adipocyte metabolism. LCFAs present within adipocytes can enhance adipose thermogenesis [21–23] and trigger inflammation in adipose tissue [24, 25]. However, the role of ELOVL6 and LCFAs in CAC-induced fat loss remains unclear.

In summary, it is reasonable to hypothesize that ELOVL6 could be involved in fat loss in CAC. This study aimed to investigate both the expression level and function of ELOVL6 in the process of fat loss in CAC. The present study revealed that the expression of ELOVL6 was downregulated in the WAT of CAC patients. A low expression of *ELOVL6* was linearly correlated with the extent of body mass reduction. Finally, this study revealed that ELOVL6 may facilitate CAC-induced fat loss by altering the fatty acid composition of adipocytes.

## Methods

### Patients and tissue samples

This study recruited patients who received diagnoses at the General Surgery Department of Zhongshan Hospital, Fudan University, during the period spanning from July 2019 to June 2020. The inclusion criteria were as follows: (1) confirmation of gastric or colorectal cancer through a pathological assessment; (2) underwent radical surgery at the hospital; and (3) available complete clinical data. The exclusion criteria were as follows: (1) previously received a preoperative anticancer treatment (chemotherapy, targeted therapy, immunotherapy, or radiotherapy); (2) presence of severe organ dysfunction (renal failure, liver insufficiency, heart failure or chronic obstructive pulmonary disease), immune impairment (autoimmune disorders, lymphoma, leukemia or acquired immune deficiency syndrome) or uncontrolled metabolic syndrome (hyperthyroidism or diabetes). All enrolled patients were briefed about the research and consented to participate.

Clinical data (age, sex, height, weight, weight loss, tumor site, and tumor stage) for the enrolled patients were recorded. Nutrition-related laboratory parameters, including hemoglobin, albumin, prealbumin, and total protein levels, were also recorded.

According to the American Society of Clinical Oncology (ASCO) guidelines on CAC management [2, 4], patients were defined as having CAC if they satisfied one or more of the following conditions: (1) a decrease in body weight exceeding 5% during the preceding 6 months or (2) a weight reduction of more than 2% coupled with a body mass index (BMI) less than 20 kg/m^2^.

Abdominal subcutaneous WAT was carefully separated and divided into approximately 500 mg pieces before being transferred to sample storage tubes. The samples were then rapidly frozen using liquid nitrogen and preserved at -80 °C for subsequent examination.

### CAC rodent model

Male C57BL/6 mice weighing 18–20 g and aged 6–7 weeks were randomly assigned to two study groups: (1) the healthy control group (CONT) and (2) the tumor-bearing group (CAC). The mice were provided by the Shanghai Institutes for Biological Science. The CAC mice were subcutaneously injected with 5*10^6^ Lewis lung carcinoma (LLC) cells dissolved in phosphate-buffered saline (PBS) near their ilium in the lower back. In contrast, the CONT group received PBS injections only. All mice were euthanized 28 days after LLC cell or PBS injection. Samples of inguinal white adipose tissue (iWAT), epididymal white adipose tissue (eWAT), gastrocnemius muscle, liver, and heart tissue were collected from each mouse. The samples were then rapidly frozen using liquid nitrogen and preserved at -80 °C for subsequent examination.

### RNA sequencing

Total RNA extraction was performed using the TRIzol reagent (Thermo Fisher, China, No. 15596018CN) according to the official guidelines. The degradation and contamination of RNA were identified using agarose gel (1%) electrophoresis. The RNA concentration was determined by employing a NanoPhotometer spectrophotometer (Implen, Germany) at OD260/280 and OD260/230. RNA integrity was assessed by an Agilent 2100 Bioanalyzer (Agilent, USA). Subsequently, mRNA was extracted and constructed into a cDNA library by NEB kits following official guidelines (NEB, USA, No. #E7490 and #E7775). After assembly, the library was quantified with a Qubit 2.0 fluorometer (Thermo Fisher, USA) and then diluted to a concentration of 1.5 ng/µL using enzyme-free water. The insert fragments were detected by using an Agilent 2100 Bioanalyzer. The libraries were quantified using qRT‒PCR while ensuring that the effective library concentration exceeded 2 nM. Sequencing of the library was completed through the Illumina HiSeq 4000 platform.

### Bioinformatic data processing

The raw data in fastq format underwent initial processing and data cleaning with Perl scripts developed in-house. After that, the read number of each gene was counted and normalized. The reference genome and its corresponding index were assembled using HISAT2 v2.0.5 after being downloaded from the Ensembl database (Ensembl *Mus musculus* release-94). Differential expression analysis was performed between two sets of data utilizing the DESeq2 R package (version 1.16.1). Genes identified as differentially expressed had an adjusted *p* value (padj) less than 0.05. The Gene Ontology (GO) database (version 2020-04-23, 10.5281/zenodo.3765910) [26, 27] and Kyoto Encyclopedia of Genes and Genomes (KEGG) database (release 94.0) [28] were used for enrichment analysis. Among the genes with differential expression, GO terms and KEGG pathways that demonstrated a padj less than 0.05 were deemed significantly enriched. The mRNA-seq dataset used in this study has been uploaded to the Gene Expression Omnibus database (GSE242812).

### Real-time PCR

Total RNA from both animal and human tissue was extracted by using the TRIzol reagent (Thermo Fisher, China, No. 15596018CN). Reverse transcription of mRNAs was conducted by employing a Qiagen Quantinova kit (Qiagen, China, No. 205,411). A real-time PCR system was established with a Qiagen SYBR Green PCR Kit (Qiagen, China, No. 208,052), and the reactions were performed on a LightCycler 480 platform (Roche, China). The list of PCR primers used is provided in the supplemental materials.

### Western blotting

RIPA lysis buffer (Thermo Fisher, China, No. 89,901) was used for protein extraction from both tissue and cell samples, after which the proteins were quantified by a BCA protein assay kit (Thermo Fisher, China, No 23,227). Proteins were separated by SDS‒PAGE and transferred to a nitrocellulose membrane through standard procedure. In this study, primary antibodies against β-tubulin (CST, USA; 1:1000, No. 86,298 S) and ELOVL6 (Novus Biologicals, USA; 1:1000, No. NBP2-81714) were used. Goat anti-rabbit IgG marked with horseradish peroxidase (Thermo Fisher, China, 1:30000, No. 31,460) was used as secondary antibody. A Tanon 5200 imaging system was used to identify and analyze the stained bands.

### FAME analysis

A mixture of ether and petroleum ether was used to extract fatty acids. The fatty acids were esterified to form fatty acid methyl esters (FAMEs) before being injected into the gas chromatograph for flame ionization detection. The percentage of a particular fatty acid was determined by the ratio of the peak area of that fatty acid to the sum of the peak areas of all fatty acid components.

### Cell culture

3T3-L1 mouse preadipocytes were cultivated in Dulbecco’s modified Eagle’s medium supplemented with high glucose (HG-DMEM, Thermo Fisher, China, No. 10,569,010), to which 10% neonatal calf serum (NCS, Thermo Fisher, China, No. 16,010,159) and 1% penicillin-streptomycin solution (PSS, Thermo Fisher, China, No. 15,140,122) were added. Similarly, LLC and CT26 mouse colon cancer cell lines were cultured in HG-DMEM supplemented with 10% fetal bovine serum (FBS, Thermo Fisher, China, No. 10,091,148) and 1% PSS. The cells were kept in a microbiological incubator at 37 °C with a 5% CO_2_ gas level. The Cell Bank at the Chinese Academy of Sciences generously provided all the cells.

### Differentiation of 3T3-L1 cells

For the initial differentiation of 3T3-L1 cells, differentiation-induced medium (DIM-I) consisting of DMEM supplemented with 1 µM dexamethasone (Sigma, China, No. D4902), 0.5 mM 3-isobutyl-1-methylxanthine (IBMX, Sigma, China, No. I5879), 1 µg/mL insulin (Millipore, China, No. 407,709), 1% PSS, and 10% FBS was used. Subsequently, another differentiation-induction medium (DIM-II) was used to maintain the differentiation environment. DIM-II was composed of DMEM supplemented with 1 µg/mL insulin, 1% PSS, and 10% FBS. The 3T3-L1 cell differentiation process commenced two days after the cells reached 100% confluency (D0) by switching to DIM-I medium. On Day 2 (D2), the medium was replaced with DIM-II, which was renewed every 2 days until further intervention.

### Preparation and use of conditioned medium (CM) from CT26 tumor cells

The preparation of CM from tumor cells has been described in our previous work [29]. Two days after CT26 cells (or 3T3-L1 cells) were passaged, the medium supernatant was collected and filtered. The CM consisted of 66% CT26 medium supernatant and 33% complete medium and was replaced on D4 in the CM group to form a cachexic environment. A mixture of 66% 3T3-L1 medium supernatant and 33% complete medium served as the corresponding intervention for the negative control (NC) group. Interleukin-6 (IL-6, Sangon, China, No. D111466) was added to the complete medium as a positive control at a final concentration of 100 ng/ml [30].

### Cell transfection

In 6-well plates, 3T3-L1 cells were cultured until they reached a confluency of 70–90%. Reduced-serum medium (Opti-MEM, Thermo Fisher, China, No. 31,985,062) and Lipofectamine 2000 (Thermo Fisher, China, No. 11,668,019) was used for transfection, following the official guideline. The specific siRNA was transferred into 3T3-L1 cells for the *Elovl6* KD group. The negative control group received the same procedure of transfection but with a non-targeting siRNA (Silencer™ negative control 4# siRNA, Invitrogen, China, No. AM4642). After 8 h of transfection, the medium was replaced with complete medium. The cells were allowed to grow until they reached 100% confluence for differentiation, as previously described. Sangon Biotech (Shanghai, China) custom-designed and produced the siRNAs used for *Elovl6* knockdown. It features a sense strand sequence of GCUCUUCGAACUGGUGCUUTT and an antisense strand sequence of AAGCACCAGUUCGAAGAGCTT.

### Oil red O (ORO) staining

The ORO solid powder (Sigma, China, No. O0625) was dissolved in isopropanol to a concentration of 0.5% and then filtered with filter paper to obtain the ORO staining solution. It was stored in the dark at 4 °C. After being prepared for use, the storage solution was diluted 60% in water and filtered with filter paper to obtain the ORO staining working solution. The cells underwent two rounds of PBS rinsing, followed by fixation with 4% paraformaldehyde at room temperature (RT) for half an hour. Subsequently, they received another round of washing with diluted water. Then, the cells were subjected to ORO staining solution and allowed to stain for 30 min at RT. Afterward, destaining was carried out by a single wash with absolute ethanol. After staining each well, 1 ml of isopropanol was added, and the absorbance was measured at 510 nm with a TECAN spectrophotometer.

### Statistical analysis

Statistical hypothesis testing and plotting were performed using GraphPad Prism 9.0.0. Normality testing was conducted using both the Anderson–Darling and Shapiro–Wilk tests. Based on the normality of the data, Student’s t test or the Kruskal‒Wallis rank-sum test was used to evaluate overall mean differences between groups. The chi-squared test was used to compare the proportions of categorical variables between groups.

## Results

### Transcriptional sequencing revealed low expression of *Elovl6* in the WAT of CAC mice

**Fig. 1:**
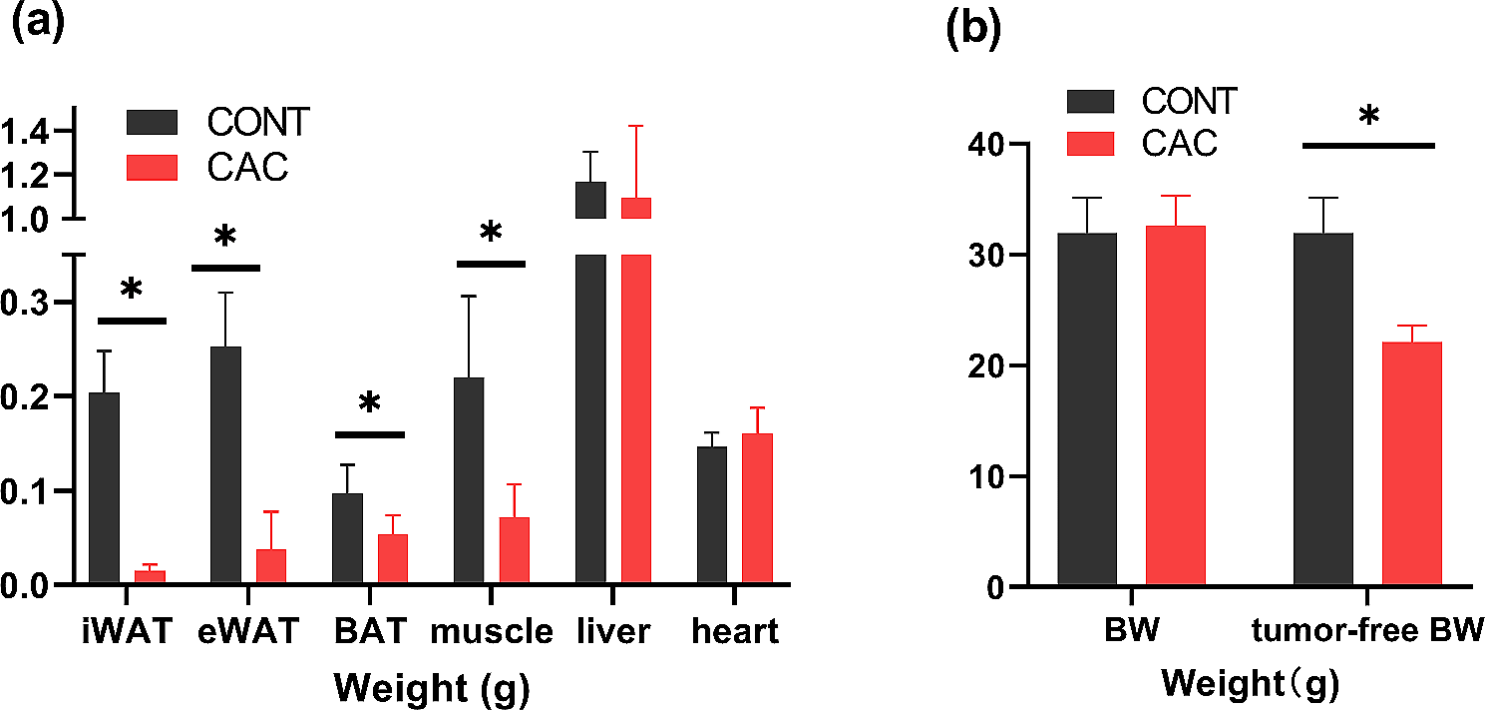
Change of body composition and weight in CAC mice. (**a**). Comparison of fat, muscle, liver, and heart weights between CAC (*n* = 10) and control (*n* = 10) mice. (**b**). Comparison of body weight (BW) and tumor-free BW between CAC and control mice. The findings are presented as the mean ± SEM, with statistical significance indicated by *P < 0.05

First, a CAC rodent model was established by subcutaneously injecting LLC cells into mice, and mice injected with PBS served as the control group (CONT). Four weeks after LLC cell injection, mice in the CAC group exhibited a notable reduction in tumor-free body weight and exhibited fat and muscle atrophy in multiple locations (Fig. 1a and b), indicating the successful establishment of this CAC rodent model.

**Fig. 2:**
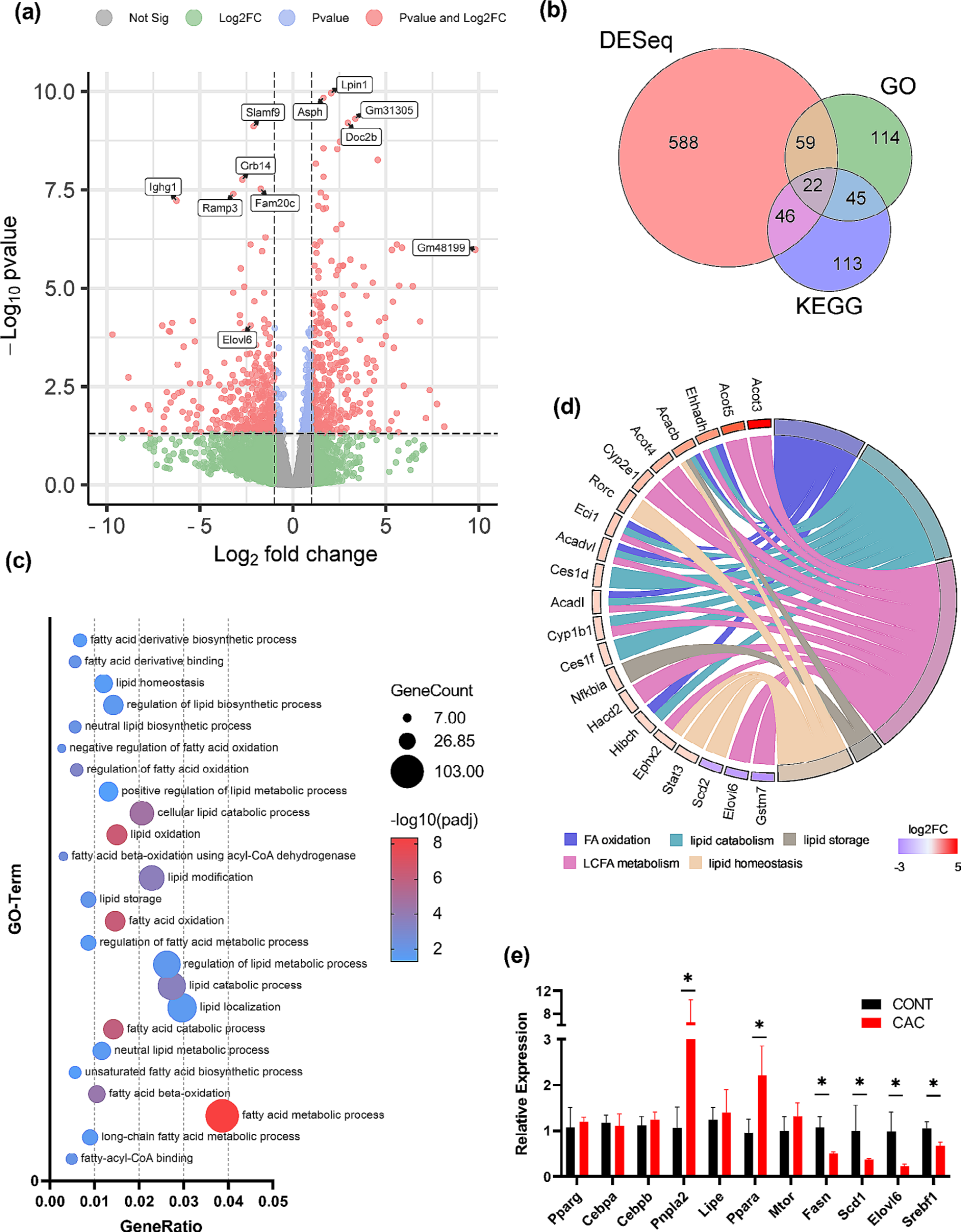
Screening for differentially expressed genes in CAC model and CONT mice. (**a**). Volcano plot illustrating the genes with differential expression according to mRNA sequencing. (**b**). Venn diagram showing the common DEGs and genes included in the GO and KEGG enrichment analyses. (**c**). Twenty-five significant GO terms related to fatty acid metabolism. (**d**). Chord diagram of 22 function-annotated genes. (**e**). qPCR analysis of the relative mRNA expression levels of genes associated with WAT hydrolysis, synthesis, and adipose neogenesis in CAC and control mice. The findings are presented as the mean ± SEM, with statistical significance indicated by *P < 0.05

Next, eWATs were obtained from 6 mice for transcriptome sequencing, comprising 3 from the CAC group and 3 from the CONT group. In total, 31,447 genes were successfully identified. A total of 715 genes were significantly differentially expressed in the WAT of CAC mice according to the criteria for differential expression (|log2(fold change)| greater than 1 and padj less than 0.05). Among these genes, 368 were upregulated, while 347 were downregulated (Fig. 2a). The GO and KEGG databases were used for enrichment analysis. A total of 328 GO terms exhibited significant enrichment, including 25 GO terms related to fatty acid and lipid metabolism (Fig. 2c). Moreover, 19 significantly enriched KEGG pathways were identified (Supplemental Fig. S1). A total of 22 genes related to FA metabolism were identified by combining the results of GO and KEGG enrichment analysis (Fig. 2b). Thirteen genes related to LCFA metabolism were identified (Fig. 2d). The functions of these genes were subsequently reviewed, as shown in Table 1 [16, 17, 31–40]. Notably, the expression of *Elovl6* was significantly downregulated in the WAT of CAC mice (log2FC = -2.27, padj = 0.0000897). This result was validated through qRT‒PCR analysis of WAT from CAC and CONT mice (Fig. 1c). The data indicated a significant downregulation of *Elovl6* and its upstream regulatory gene *Srebf1*. Therefore, *Elovl6* is the gene with the most potential for subsequent research.

**Table 1:** Thirteen DEGs related to LCFA metabolism in the WAT of CAC mice

Gene	Log2(FC)	Padj	Function
*Cyp2e1*	1.769	< 0.0001	Encodes a cytochrome P450 protein involved in omega hydroxylation of PUFAs [31]
*Acot3*	5.374	< 0.0001	Catalyzes the hydrolysis of long-chain acyl-CoAs [32]
*Eci1*	1.463	< 0.0001	Involved in beta-oxidation of unsaturated fatty acids [33]
*Elovl6*	-2.273	< 0.0001	Catalyzes carbon chain lengthening of C12-C16 fatty acids [16, 17]
*Hacd2*	1.095	0.0009	Catalyzes the third step of LCFA carbon chain extension (dehydration) [34]
*Acot4*	1.983	0.0014	Catalyzes the hydrolysis of long-chain acyl-CoAs [32]
*Ephx2*	1.064	0.0049	Degrades epoxides and arene oxides [35]
*Ehhadh*	2.844	0.0049	Involved in the peroxisomal β-oxidation pathway [36]
*Acadvl*	1.448	0.0106	Catalyzes the initial stage of VLCFA mitochondrial fatty acid β-oxidation [37]
*Acadl*	1.205	0.0114	Catalyzes the initial stage of LCFA mitochondrial fatty acid β-oxidation [38]
*Gstm7*	-2.580	0.0124	Predicted to be involved in LCFA metabolism [39]
*Acot5*	4.266	0.0355	Catalyzes the hydrolysis of long-chain acyl-CoAs [32].
*Cyp1b1*	1.171	0.0355	Encodes a cytochrome P450 protein involved in metabolism of multiple PUFAs [31, 40]

### Low expression of ELOVL6 in the WAT of CAC patients could change the fatty acid proportion and worsen weight loss

To validate the decreased expression of ELOVL6 in the WAT of CAC patients, 204 subjects who met the aforementioned inclusion and exclusion criteria were included in our study. Thirteen patients were excluded due to metabolic diseases and preoperative neoadjuvant chemotherapy. Ultimately, the study included 191 patients. In total, 99 CAC patients and 92 weight-stable cancer patients (WS), which served as the control group, were included in this study. The fundamental clinical information of the cancer patients in both groups is presented in Table 2. Except for weight loss and BMI, other factors, such as sex, age, and tumor node metastasis classification (TMN), were not significantly different between the CAC and WS groups, indicating comparable baseline matching between the two groups.

**Table 2:** Clinical information of the included CAC patients and weight-stable tumor patients

Features	CAC (*n* = 99)	WS (*n* = 92)	*P*
Gender, Female (n, %)	36, 36.36%	40, 43.47%	0.315
Age (yr)	62.12 ± 11.90	62.10 ± 9.47	0.991
Height (cm)	163.04 ± 7.88	166.09 ± 7.85	0.008
Weight (kg)	55.88 ± 9.62	67.46 ± 9.45	< 0.0001
BMI (kg/m^2^)	20.96 ± 2.86	24.39 ± 2.55	< 0.0001
Weight loss (kg)	4.99 ± 3.32	0.38 ± 0.91	< 0.0001
Stage, I - II (n, %)	75, 75.75%	62, 67.39%	0.199
Albumin (g/L)	39.75 ± 4.31	40.78 ± 4.24	0.421
Prealbumin (mg/L)	189.90 ± 52.14	223.58 ± 50.67	0.022
Total protein (g/L)	64.38 ± 5.76	66.29 ± 4.91	0.014
LDL (mmol/L)	2.44 ± 0.88	2.45 ± 0.82	0.959
HDL-CH (mmol/L)	1.13 ± 0.41	1.13 ± 0.36	0.937
Total cholesterol (mmol/L)	3.91 ± 0.29	4.26 ± 0.93	0.852
Triglyceride (mmol/L)	1.17 ± 0.14	1.52 ± 0.73	0.018
RBC (10^12/L)	3.89 ± 0.62	4.21 ± 0.53	0.0002
WBC (10^9/L)	5.18 ± 1.72	5.66 ± 1.90	0.06
Hb (g/L)	115.65 ± 20.32	126.91 ± 19.41	0.0001
PLT (10^9/L)	220.56 ± 71.90	218.88 ± 74.53	0.874
HBA1C (%)	5.71 ± 1.42	5.90 ± 0.88	0.246

**Fig. 3:**
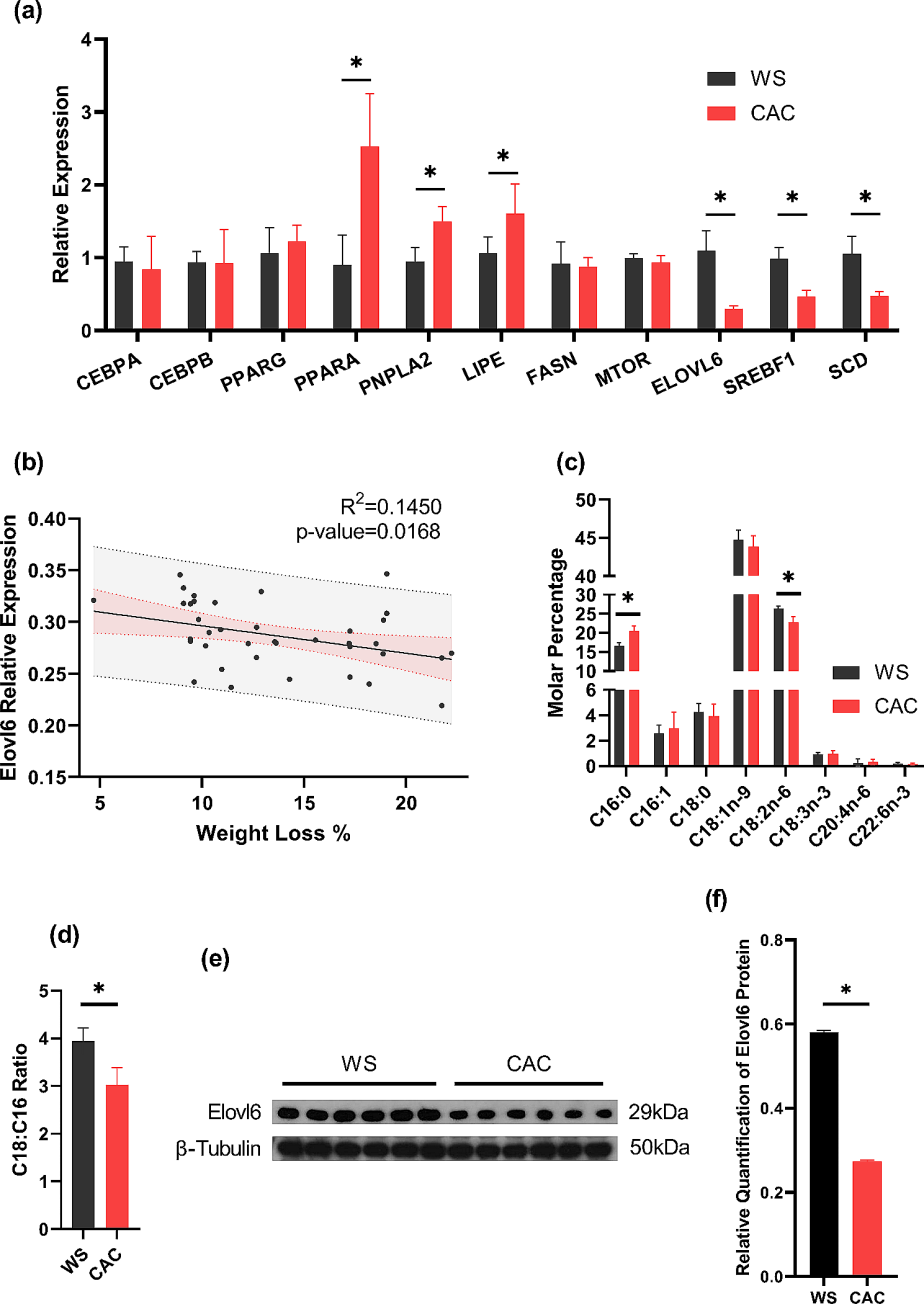
Downregulation of *ELOVL6* in the WAT of CAC patients may alter the fatty acid proportion and exacerbate weight loss. (**a**). qPCR analysis of genes associated with lipid metabolism in the WAT of weight-stable tumor patients (WS) and CAC patients. (**b**). Correlation analysis between *ELOVL6* expression in WAT and the degree of weight loss in CAC patients. (**c**). and (**d**). Fatty acid analysis of WAT from the WS group and CAC group, showing the composition of different fatty acids (**c**). and C18:C16 ratio (**d**). between the two groups. (**e**). and **f.** Western blot analysis of ELOVL6 in both the CAC and WS groups. The findings are presented as the mean ± SEM, with statistical significance indicated by *P < 0.05

In both groups of patients, the qPCR results for genes associated with lipid metabolism in WAT demonstrated a notable downregulation of *ELOVL6* and its upstream regulatory gene, *SREBF1*. The expression levels of genes related to lipolysis, including hormone-sensitive lipase (*LIPE*), peroxisome proliferator-activated receptor alpha (*PPARA*), and patatin-like phospholipase domain containing 2 (*PNPLA2*), were significantly increased in the WAT of CAC patients. In contrast, there were no notable differences in the expression of genes associated with adipogenesis, including enhancer-binding protein alpha (*CEBPA*), *CEBPB*, and *PPARG*, or lipogenic genes, such as mammalian target of rapamycin (*MTOR*) and fatty acid synthase (*FASN*) (Fig. 3a). Moreover, there was a significant downregulation of the expression of the gene stearoyl-CoA desaturase (*SCD*), which is implicated in the desaturation of stearoyl-CoA. Western blotting also revealed a decrease in ELOVL6 protein expression in CAC patients (Fig. 3e and f). Then, a correlation analysis of *ELOVL6* expression with the degree of weight loss in CAC patients was performed (Fig. 3b), and the results suggested a statistically significant linear relationship between the two variables.

To identify alterations in fatty acid content in the WAT of CAC patients, fatty acids were isolated from tissue samples and converted into FAMEs that were suitable for GC analysis. The content of palmitic acid (PA, C16:0) was significantly greater in the WAT of CAC patients. Similarly, the content of linoleic acid (LA, C18:2n-6) was significantly lower (Fig. 3c). When comparing the overall C18 to C16 fatty acid composition between the two groups, it became evident that there was a notable reduction in the C18:C16 ratio within the WAT tissues of CAC patients (Fig. 3d). There were no significant variations observed in the relative contents of other fatty acids (Supplemental Table S2).

### *Elovl6* knockdown inhibited lipogenesis and preadipocyte differentiation by altering fatty acid components in adipocytes in vitro

**Fig. 4:**
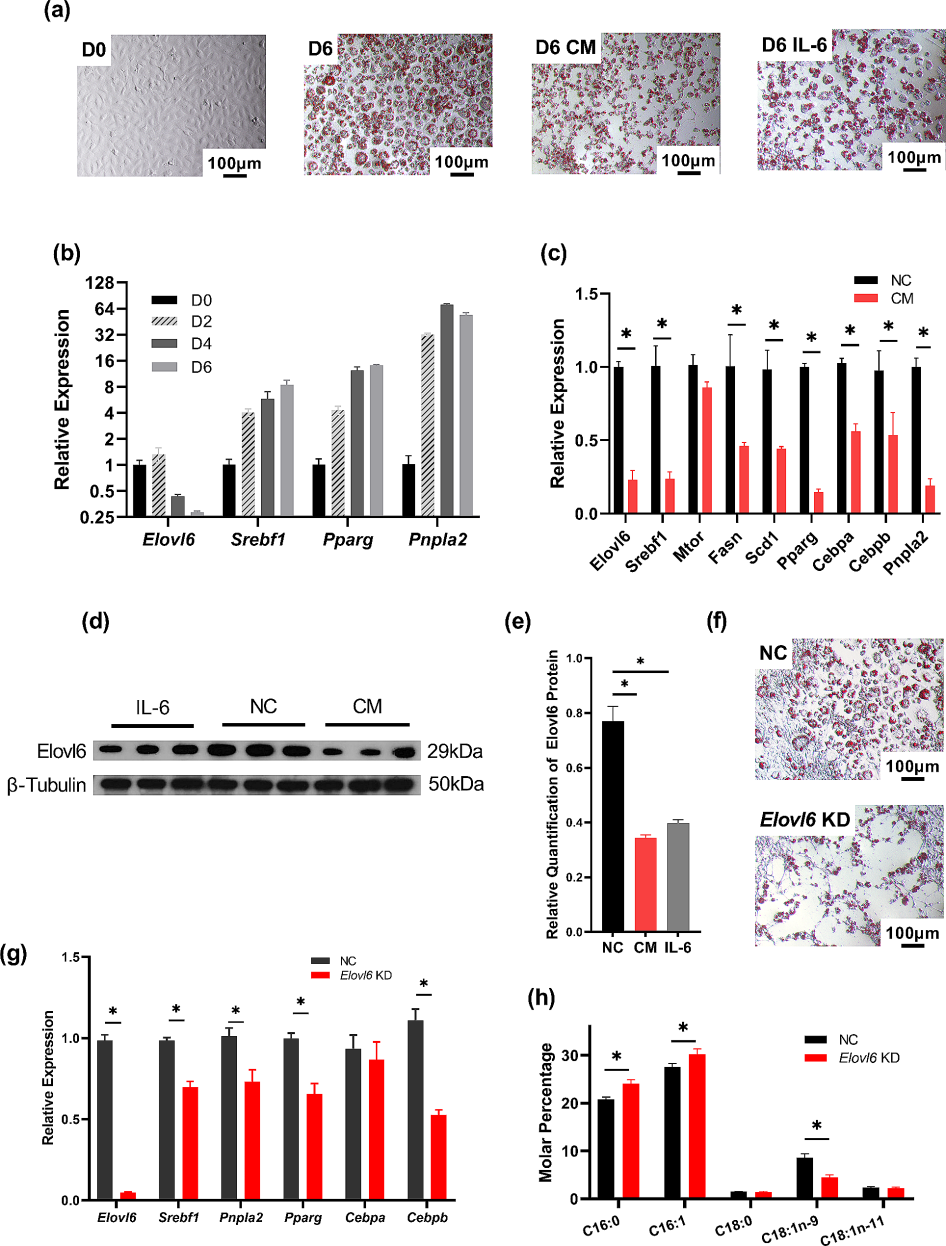
Elovl6 knockdown inhibits preadipocyte differentiation and affects adipocyte fatty acid metabolism. (**a**). Micrographs of ORO staining in 3T3-L1 cells before differentiation (D0), 6 days after differentiation (D6), and with conditioned medium from CT26 tumor cells added after differentiation (D6 CM). The IL-6-treated group was used as a positive control (D6 IL-6). (**b**). Changes in the expression of genes associated with lipid metabolism and adipogenesis were observed in 3T3-L1 cells at various time points following differentiation. (**c**). Alterations in the relative expression of genes associated with lipid metabolism were observed in 3T3-L1 cells following differentiation when exposed to the conditioned medium (CM) or a control (NC). (**d**). and (**e**). Western blot analysis of Elovl6 in the CM, NC, and IL-6 groups. (**f**). Micrographs of ORO staining in differentiated 3T3-L1 cells after exposure to the CM and treatment with *Elovl6* siRNA (Elovl6 KD) or the control (NC) were acquired. (**g**). Changes in the expression of genes related to lipid metabolism in differentiated 3T3-L1 cells treated with CAC-conditioned medium and treated with *Elovl*6 siRNA (*Elovl6* KD) or the control (NC). (**h**). Fatty acid analysis of differentiated 3T3-L1 cells following treatment with *Elovl6* siRNA (*Elovl6* KD) or the control (NC) was performed using gas chromatography. The findings are presented as the mean ± SEM, with statistical significance indicated by * *P* < 0.05

To further explore the influence of *Elovl6* on the morphology and function of adipocytes, a tumor cell-conditioned medium was used to differentiate 3T3-L1 cells and establish an in vitro model of CAC. Complete medium supplemented with IL-6 served as a positive control. Additionally, siRNA was used to suppress *Elovl6* expression in 3T3-L1 preadipocytes, and a non-targeting siRNA was used as a control. Following the onset of differentiation in 3T3-L1 cells, the preadipocytes underwent gradual transformation into a full, round shape, with a concurrent increase in both the quantity and diameter of intracellular lipid droplets (Fig. 4a). In 3T3-L1 cells, the qPCR results indicated a decrease in *Elovl6* expression during differentiation, while the expression of the upstream regulatory gene of *Elovl6*, *Srebf1*, progressively increased. Additionally, *Pparg*, a gene related to differentiation, and *Pnpla2*, a major gene associated with lipolysis, were also gradually upregulated (Fig. 4b). After 3T3-L1 cells differentiated and matured, conditioned medium from CT26 tumor cells (CM) was added to simulate a cachexic environment. In the CM group, the adipocyte morphology underwent significant changes, including a reduction in both the quantity and volume of intracellular lipid droplets (Fig. 4a). qPCR assays indicated a decrease in the expression level of genes related to the differentiation process, such as *Cebpa*, *Cebpb*, and *Pparg*, as well as genes associated with lipid synthesis, including *Mtor*, *Fasn*, and *Scd-1*. The expression levels of *Elovl6* and *Srebf1* were also notably reduced (Fig. 4c). Western blot analysis also revealed a decrease in Elovl6 protein expression in the CAC group (Fig. 4d and e).

After using siRNA to knock down *Elovl6* expression in 3T3-L1 cells, the volume of lipid droplets in the cytoplasm after differentiation was also reduced, as observed through ORO staining in Fig. 4f. qPCR analysis revealed that silencing *Elovl6* suppressed the expression of genes associated with fatty acid synthesis, hydrolysis, and adipogenesis (Fig. 4g), including *Srebf1*, *Pnpla2*, *Pparg*, and *Cebpb*. Mature 3T3-L1 cells were then collected from both the *Elovl6*-KD and control groups, and their fatty acid composition was analyzed by GC, indicating that the knockdown of *Elovl6* in 3T3-L1 cells could cause alterations in fatty acid composition. This was evidenced by a notable increase in the content of PA and a decrease in the content of OA (Fig. 4h). These results suggested that low expression of Elovl6 may exaggerate tumor cell-induced adipocyte atrophy, probably through altering the fatty acid component of adipocytes.

## Discussion

In this study, transcriptome sequencing of the WAT of the CAC rodent model was performed. A significant decrease in ELOVL6 expression was observed in CAC patients and CAC mice. This result was verified by an in vivo study using a rodent CAC model and additional experiments using clinical CAC samples. The reduced expression of *ELOVL6* was also correlated with the degree of weight loss in CAC patients, which further corroborates the relationship between ELOVL6 and pathophysiological processes in CAC.

The first question that comes to mind is whether the low expression of ELOVL6 in the WAT of CAC patients could be a consequence of altered fatty acid metabolism in the pathophysiological process of CAC. Currently, no studies have reported the expression and regulation of ELOVL6 in CAC. In this study, the expression of both ELOVL6 and SREBP1 was decreased in CAC patients, tumor-bearing mice, and 3T3-L1 cells in a cachexic environment. Other related studies have suggested that SREBP1 can regulate ELOVL6 expression. Moon and colleagues observed high expression of Elovl6 in transgenic mice overexpressing Srebp1 [15]. Furthermore, synthetic agonists of *LXR*, an upstream regulatory gene of SREBP1, induced high expression of ELOVL6 and FASN in BAT adipocytes [41]. These reports revealed the positive regulatory effect of SREBP1 on ELOVL6. SREBP family proteins are inactivated precursor proteins anchored to the endoplasmic reticulum (ER) membrane. Upon the formation of their structural domains, SREBPs can regulate both cholesterol and fatty acid metabolism [42, 43]. According to relevant studies, the anabolic AKT-MTOR signaling pathway could upregulate SREBP1 [44, 45]. Thus, it is possible that the downregulation of ELOVL6 expression in the WAT of CAC patients could be a phenomenon following the inhibition of the entire lipid anabolic process.

Notably, no alterations in *MTOR* mRNA expression were observed in either the CAC rodent model or clinical samples. In fact, in cancer-associated cachexia, decreased *MTOR* expression has been described exclusively in the skeletal muscle of CAC patients or CAC mice, with no such reports in adipose tissue [46–48]. However, this study revealed downregulation of *SREBF1*, *FASN*, and *SCD* expression in the WAT of CAC patients, indicating downstream inhibition of the fatty acid synthesis pathway. In other studies, the mRNA levels of genes related to adipose synthesis, including *FASN*, *LIPE*, and *SCD*, were also reduced in the adipose tissue of tumor-bearing mice [49]. These results suggested that the downregulation of SERBP1 in the WAT of CAC patients may be induced by a change in the phosphorylation of MTOR and could trigger downstream inhibition of lipid synthesis [50].

**Fig. 5:**
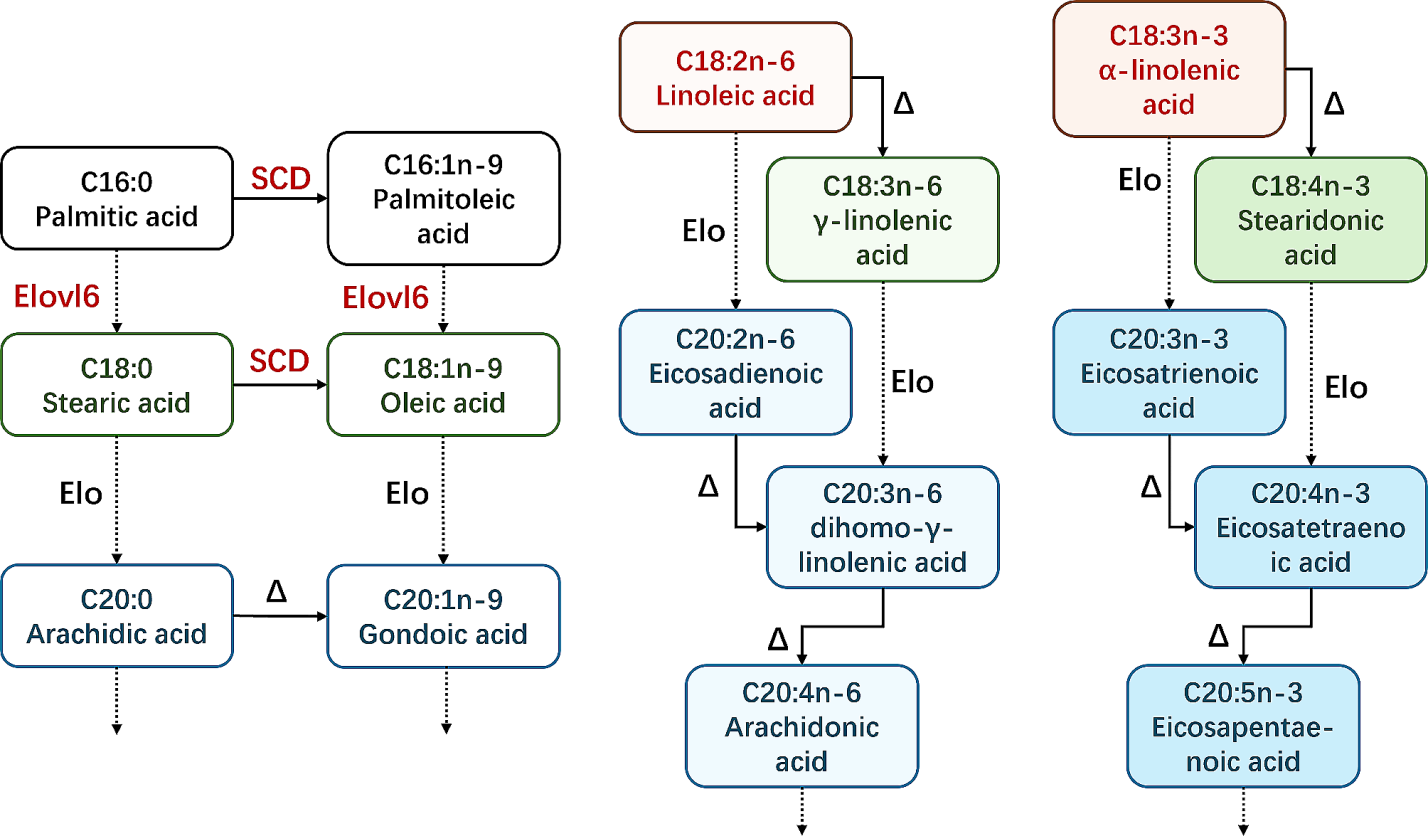
Biosynthetic pathways of LCFAs in Homo sapiens. Elongation reactions are indicated by dashed lines labeled Elo, while desaturation reactions are indicated by solid lines labeled Δ

Thus, it is reasonable to assume that ELOVL6 is downregulated by the inhibition of lipid synthesis during CAC pathophysiology, possibly through the MTOR-SREBP axis. On the other hand, the low expression of ELOVL6 may also contribute to fat loss. In this study, the knockdown of *Elovl6* suppressed the differentiation of preadipocytes and altered lipid metabolism. Additionally, PA content in the WAT of CAC patients was increased, while LA content was decreased. After the knockdown of *Elovl6* in 3T3-L1 cells, a similar change in the fatty acid composition of the cells occurred. A significant increase in the PA content and a decrease in the OA content were observed in the *Elovl*6-KD groups in vitro. This phenomenon not only matches the decreased expression of ELOVL6 but also implies that ELOVL6 might have a subsequent biological impact by altering the proportion of monounsaturated fatty acids (MUFAs) in WAT. Kobayashi et al. reported that adding C18:1 and C20:1 FAs to the culture media of 3T3-L1 cells could facilitate differentiation [51], which may support this hypothesis. Similarly, Malodobra-Mazur et al. found that OA could inhibit methylation of *Pparg* and *Cebpa* promoters, resulting in increased differentiation of 3T3-L1 cells [52]. Since the biosynthesis of MUFAs requires long-chain acyl-CoAs as substrates and ELOVL6 is a crucial enzyme in LCFA synthesis within WAT, the decreased expression of ELOVL6 in the WAT of CAC patients may further regulate the process of fat loss during CAC by affecting the synthesis of MUFAs (Fig. 5).

Interestingly, the decrease of LA in the WAT of CAC patients was also found in our study. LA is an essential fatty acid that cannot be synthesized in mammals due to the absence of the corresponding desaturase [53]. Indeed, the regulatory influence of fatty acids on lipid metabolism has been a long-standing research topic, particularly regarding polyunsaturated fatty acids (PUFAs). A study reported that feeding PUFAs to mice inhibited FASN expression and related lipid anabolism [54]. Worgall et al. reported that oleic acid and PUFAs could inhibit SREBP expression, ultimately leading to the inhibition of fatty acid synthesis [55]. In recent years, Ou et al. reported that PUFAs could directly inhibit the expression of *LXR*, an upstream regulatory gene of SREBP [41]. These studies suggest that PUFAs have an inhibitory effect on lipid synthesis pathways. However, no such change in PUFAs other than LA was observed either in the WAT of CAC patients or in *Elovl6*-KD 3T3-L1 cells. We believe that the primary reason for the lack of positive results is the low amount of PUFAs in WAT and 3T3-L1 cells. Thus, additional evidence is still required to support this viewpoint.

### Comparisons with other studies and what does the current work add to the existing knowledge

Currently, no studies have been conducted on ELOVL6 and other fatty acid elongases in CAC. However, Tan et al. reported a significant reduction in the C18:C16 ratio and a decrease in the expression of *SCD* family genes in the WAT of *Elovl6* KO mice, which indicates that the fatty acid desaturation process was suppressed [20]. Interestingly, the WAT of CAC patients in this study exhibited similar alterations. In this study, transcriptome sequencing was conducted on WAT from CAC mice, and fatty acid GC analysis was performed on WAT from CAC patients. The resulting data provide valuable insights for future studies on CAC-induced fat loss. Additionally, the current study proposed the potential contribution of ELOVL6 to the process of CAC-induced fat loss. This discovery not only presents a new biomarker for diagnosing and treating CAC in future clinical practice but also introduces a new theory for CAC research.

### Study strengths and limitations

This study is closely relevant to the clinical field. First, most WAT samples used in this study were obtained from CAC patients diagnosed by clinical doctors, which makes the results representative of real situations in the human body. Furthermore, both a decrease in ELOVL6 expression and a change in the fatty acid composition were detected in the fat tissue of CAC patients, indicating that ELOVL6 could be a valuable biomarker for the early identification of CAC. Finally, Elovl6 altered the fatty acid composition in adipocytes in vitro, suggesting that it may contribute to fat loss in CAC. Therefore, ELOVL6 is a promising target for future therapeutic interventions. A limitation of our research was that *Elovl6* knockdown was performed in vitro, which may not reflect the actual conditions in the human body.

## Conclusion

Overall, ELOVL6 expression was decreased in the WAT of CAC patients. Low expression of ELOVL6 correlated with the severity of weight loss in patients with CAC. In addition, reduced ELOVL6 expression in the WAT of CAC patients coincided with a reduction in C18 fatty acid content and an increase in C16 fatty acid content. Moreover, *Elovl6* knockdown inhibited preadipocyte differentiation and downregulated the expression of genes associated with lipid synthesis. *Elovl6* knockdown also induced a significant decrease in the oleic acid (C18:1n-9) content and a notable increase in the palmitic acid (C16:0) content, suggesting that low expression of ELOVL6 could suppress the CAC liposynthesis process possibly by altering the lipid content in adipocytes. The findings from this study indicate that ELOVL6 has the potential to serve as a reliable biomarker for the early diagnosis of cancer-associated cachexia and may be a promising target for future therapies.

### Electronic supplementary material

Below is the link to the electronic supplementary material.

Additional file 1: Table S1: List of qPCR primers; Table S2: Fatty acid profile of CAC patients and control patients; Table S3: Fatty acid profile of *Elovl6*-KD and NC mature 3T3-L1 adipocytes. Figure S1: Top 20 significant KEGG pathways in WAT of CAC and CONT mice

## Data Availability

The mRNA-seq dataset used in this study has been uploaded to the Gene Expression Omnibus database (GSE242812).

## Abbreviations

CAC: Cancer-associated cachexia
WAT: White adipose tissue
ELOVL6: Elongase of very long-chain fatty acid 6
BAT: Brown adipose tissue
LCFA: Long-chain fatty acid
TG: Triglyceride
HDL-C: High-density lipoprotein cholesterol
LDL: Low-density lipoprotein
BMI: Body mass index
LLC: Lewis lung carcinoma
PBS: Phosphate-buffered saline
FAME: Fatty acid methyl ester
GC: Gas chromatography
NCS: Neonatal calf serum
HG-DMEM: Dulbecco’s modified Eagle’s medium with high glucose
PSS: Penicillin‒streptomycin solution
FBS: Fetal bovine serum
IBMX: 3-isobutyl-1-methyl-7 H-xanthine
ORO: Oil Red O
GO: Gene Ontology
KEGG: Kyoto Encyclopedia of Genes and Genomes
SREBF1: Steroid regulatory element-binding protein-1
PNPLA2: Patatin-like phospholipase domain containing 2
LIPE: Hormone sensitive lipase
LPL: Lipoprotein lipase
CEBP: Enhancer-binding protein
PPAR: Peroxisome proliferator-activated receptor
FASN: Fatty acid synthase
SCD: Stearoyl-CoA desaturase
