# Supplementary material for: Low expression of ELOVL6 may be involved in fat loss in white adipose tissue of cancer-associated cachexia
Source: Lipids Health Dis. 2024 May 17;23:144. doi: 10.1186/s12944-024-02126-9 (PMC11100253; doi:10.1186/s12944-024-02126-9)
Supplement: Supplementary file 1 — Additional file 1: Table S1: List of qPCR primers; Table S2: Fatty acid profile of CAC patients and control patients; Table S3: Fatty acid profile of Elovl6-KD and NC mature 3T3-L1 adipocytes. Figure S1: Top 20 significant KEGG pathways in WAT of CAC and CONT mice [file 12944_2024_2126_MOESM1_ESM.docx]

**Additional File 1**

Table S1. List of qPCR primers

| Organism | Gene | Forward Primer (5'- 3') | Reverse Primer (5'- 3') |
| --- | --- | --- | --- |
| Mus musculus | *Cebpa* | CTAGGAGATTCCGGTGTGGC | CCCGAGAGGAAGCAGGAATC |
| Mus musculus | *Cebpb* | CGCCTTATAAACCTCCCGCT | TGGCCACTTCCATGGGTCTA |
| Mus musculus | *Pparg* | CCAAGAATACCAAAGTGCGATC | TCACAAGCATGAACTCCATAGT |
| Mus musculus | *Fasn* | GTGAGACTCCTTGGAGGGTG | TCTAGAGGGCTTGCACCAAC |
| Mus musculus | *Scd1* | CGCCCCTACGACAAGAACAT | AAGCCCAAAGCTCAGCTACT |
| Mus musculus | *Mtor* | ACTGTGTCTTGGCATCCCTG | AGCCTTCAGGATAGGCTCCA |
| Mus musculus | *Pnpla2* | CAGAGATGGACTTCGATTCCTT | CAGGTGCTCTAGAATTCGATCT |
| Mus musculus | *Lipe* | GGAGCTCCAGTCGGAAGAGG | GTCTTCTGCGAGTGTCACCA |
| Mus musculus | *Elovl6* | TGCAGCATGACAACGACCAGTG | AATGGCAGAAGAGCACAAGGTAGC |
| Mus musculus | *Srebf1* | GCTACCGGTCTTCTATCAATGA | CGCAAGACAGCAGATTTATTCA |
| Mus musculus | *Ppara* | CGTTTGTGGCTGGTCAAGTT | TCCTCACCGATGGACTGAGA |
| Mus musculus | *Ucp1* | ATTCAGAGGCAAATCAGCTTTG | GTGTTTCTCTCCCTGAAGAGAA |

| Organism | Gene | Forward Primer (5'- 3') | Reverse Primer (5'- 3') |
| --- | --- | --- | --- |
| Homo sapiens | *CEBPA* | TATAGGCTGGGCTTCCCCTT | AGCTTTCTGGTGTGACTCGG |
| Homo sapiens | *CEBPB* | GGCCGGTTTCGAAGTTGATG | TGCCCCCAAAAGGCTTTGTA |
| Homo sapiens | *PPARG* | TGACCAGAAGCCTGCATTTCT | ACGGAGCTGATCCCAAAGTT |
| Homo sapiens | *FASN* | CGCGGTTTAAATAGCGTCGG | AGACAGGTCCTTCAGCTTGC |
| Homo sapiens | *SCD* | CTTGCGATATGCTGTGGTGC | CCGGGGGCTAATGTTCTTGT |
| Homo sapiens | *MTOR* | GCCGCGCGAATATTAAAGGA | CTGGTTTCCTCATTCCGGCT |
| Homo sapiens | *PNPLA2* | GGGACTCACAGTTGCCAAGA | CAAGTAAGCAGGCGGTCACA |
| Homo sapiens | *LIPE* | ATTACTTAGTGGGGGCCAGC | GGATCCCTGCAGAGTCTTCG |
| Homo sapiens | *ELOVL6* | GAGAATGAAGCCATCCAAT | CAGAGACCAGAGCACTAA |
| Homo sapiens | *SREBF1* | TTCCGAGGAACTTTTCGCCG | GCCGACTTCACCTTCGATGT |
| Homo sapiens | *PPARA* | TCACCACAGTAGCTTGGAGC | GTGAAAGCGTGTCCGTGATG |

Table S1 (Continued). List of qPCR primers

Table S2. Fatty acid profile of CAC and control patients

| Fatty acids | WS (n=12) | CAC (n=12) | p-value |
| --- | --- | --- | --- |
| C6:0 | <0.01 | <0.01 | / |
| C8:0 | <0.01 | <0.01 | / |
| C10:0 | <0.01 | <0.01 | / |
| C11:0 | <0.01 | <0.01 | / |
| C12:0 | 0.072 ± 0.015 | 0.098 ± 0.031 | 0.0833 |
| C13:0 | <0.01 | <0.01 | / |
| C14:0 | 0.87 ± 0.11 | 1.06 ± 0.31 | 0.1783 |
| C14:1n-9 | 0.061 ± 0.019 | 0.098 ± 0.079 | 0.296 |
| C15:0 | 0.095 ± 0.014 | 0.088 ± 0.0096 | 0.3752 |
| C15:1n-10 | <0.01 | <0.01 | / |
| C16:0 | 16.63 ± 0.85 | 20.57 ± 1.22 | 0.0001 |
| C16:1n-9 | 2.56 ± 0.67 | 2.99 ± 1.24 | 0.4668 |
| C17:0 | 0.19 ± 0.024 | 0.17 ± 0.039 | 0.3925 |
| C17:1n-9 | <0.01 | <0.01 | / |
| C18:0 | 4.26 ± 0.65 | 3.92 ± 0.97 | 0.489 |
| C18:1n-11 | <0.01 | <0.01 | / |
| C18:1n-9 | 44.77 ± 1.24 | 43.88 ± 1.42 | 0.2728 |
| C18:1n-9, trans | <0.01 | <0.01 | / |
| C18:2n-6 (LA) | 26.34 ± 0.66 | 22.84 ± 1.36 | 0.0002 |
| C18:2n-6, trans | <0.01 | <0.01 | / |
| C18:3n-3 (LNA) | 0.91 ± 0.14 | 0.96 ± 0.26 | 0.6368 |
| C18:3n-6 | 0.060 ± 0.025 | 0.047 ± 0.024 | 0.3654 |
| C19:0 | <0.01 | <0.01 | / |

Table S2 (Continued). Fatty acid profile of CAC and control patients

| Fatty acids | WS (n=12) | CAC (n=12) | p-value |
| --- | --- | --- | --- |
| C20:0 | 0.29 ± 0.11 | 0.31 ± 0.15 | 0.8637 |
| C20:1n11 | 0.96 ± 0.15 | 1.11 ± 0.32 | 0.3244 |
| C20:2n-6 | 0.50 ± 0.084 | 0.54 ± 0.11 | 0.5243 |
| C20:3n-3 | 0.053 ± 0.0082 | 0.062 ± 0.015 | 0.2531 |
| C20:3n-6 | 0.28 ± 0.086 | 0.29 ± 0.11 | 0.8408 |
| C20:3n-9 | <0.01 | <0.01 | / |
| C20:4n-6 | 0.26 ± 0.31 | 0.34 ± 0.21 | 0.6394 |
| C20:5n-3 (EPA) | 0.035 ± 0.011 | 0.028 ± 0.0075 | 0.2346 |
| C21:0 | <0.01 | <0.01 | / |
| C22:0 | 0.073 ± 0.040 | 0.075 ± 0.052 | 0.9518 |
| C22:5n-3 (DPA) | 0.45 ± 0.13 | 0.46 ± 0.097 | 0.8837 |
| C22:6n-3 (DHA) | 0.21 ± 0.075 | 0.16 ± 0.054 | 0.2608 |
| C23:0 | <0.01 | <0.01 | / |
| C24:0 | 0.043 ± 0.015 | 0.050 ± 0.027 | 0.6072 |
| C24:1n-15 | 0.037 ± 0.016 | 0.040 ± 0.014 | 0.7134 |

Table S3. Fatty acid profile of *Elovl6*-KD and NC mature 3T3-L1 adipocytes

| Fatty acids | NC (n=3) | *Elovl6*-KD (n=3) | p-value |
| --- | --- | --- | --- |
| C6:0 | <0.01 | <0.01 | / |
| C8:0 | <0.01 | <0.01 | / |
| C10:0 | 0.215 ± 0.024 | 0.220 ± 0.040 | 0.8610 |
| C11:0 | 0.162 ± 0.020 | 0.155 ± 0.019 | 0.6651 |
| C12:0 | 0.311 ± 0.012 | 0.287 ± 0.035 | 0.3228 |
| C13:0 | 0.791 ± 0.033 | 0.739 ± 0.057 | 0.2374 |
| C14:0 | 6.85 ± 0.55 | 5.396 ± 1.146 | 0.1198 |
| C14:1n-9 | 1.69 ± 0.36 | 1.62 ± 0.25 | 0.8016 |
| C15:0 | 14.23 ± 0.44 | 14.61 ± 0.26 | 0.2728 |
| C15:1n-10 | <0.01 | <0.01 | / |
| C16:0 | 20.85 ± 0.43 | 24.07 ± 0.80 | 0.0036 |
| C16:1n-9 | 27.62 ± 0.63 | 30.20 ± 1.15 | 0.0272 |
| C17:0 | 2.69 ± 0.31 | 2.37 ± 0.16 | 0.1828 |
| C17:1n-9 | 7.73 ± 0.18 | 7.73 ± 0.21 | 0.9911 |
| C18:0 | 1.526 ± 0.039 | 1.42 ± 0.12 | 0.2227 |
| C18:1n-9 | 8.63 ± 0.76 | 4.53 ± 0.47 | 0.0013 |
| C18:1n-11 | 2.37 ± 0.20 | 2.23 ± 0.21 | 0.4277 |
| C18:1n-9, trans | <0.01 | <0.01 | / |
| C18:2n-6, trans | <0.01 | <0.01 | / |
| C18:2n-6 (LA) | 1.242 ± 0.054 | 1.115 ± 0.050 | 0.0402 |
| C18:3n-3 (LNA) | <0.01 | <0.01 | / |
| C18:3n-6 | <0.01 | <0.01 | / |

Table S3 (Continued). Fatty acid profile of *Elovl6*-KD and NC mature 3T3-L1 adipocytes

| Fatty acids | NC (n=3) | | *Elovl6*-KD (n=3) | | p-value | |  |
| --- | --- | --- | --- | --- | --- | --- | --- |
| C19:0 | | 0.116 ± 0.015 | | 0.135 ± 0.006 | | 0.1083 | |
| C20:0 | | <0.01 | | <0.01 | | / | |
| C20:1n-11 | <0.01 | | <0.01 | | / | |  |
| C20:2n-6 | 0.044 ± 0.001 | | 0.045 ± 0.002 | | 0.4220 | |  |
| C20:3n-3 | <0.01 | | <0.01 | | / | |  |
| C20:3n-6 | 0.035 ± 0.002 | | 0.032 ± 0.002 | | 0.1686 | |  |
| C20:3n-9 | 0.372 ± 0.031 | | 0.351 ± 0.009 | | 0.3418 | |  |
| C20:4n-6 | 0.893 ± 0.030 | | 0.97 ± 0.10 | | 0.2755 | |  |
| C20:5n-3 (EPA) | 0.123 ± 0.017 | | 0.110 ± 0.006 | | 0.2817 | |  |
| C21:0 | 0.081 ± 0.007 | | 0.079 ± 0.003 | | 0.6913 | |  |
| C22:0 | <0.01 | | <0.01 | | / | |  |
| C22:1n-13 | <0.01 | | <0.01 | | / | |  |
| C22:2n-6 | 0.133 ± 0.004 | | 0.134 ± 0.004 | | 0.7017 | |  |
| C22:4n-6 | 0.093 ± 0.007 | | 0.101 ± 0.003 | | 0.1764 | |  |
| C22:5n-3 (DPA) | 0.328 ± 0.010 | | 0.307 ± 0.007 | | 0.0459 | |  |
| C22:6n-3 (DHA) | 0.567 ± 0.067 | | 0.546 ± 0.037 | | / | |  |
| C23:0 | <0.01 | | <0.01 | | / | |  |
| C24:0 | <0.01 | | <0.01 | | / | |  |
| C24:1n-15 | 0.110 ± 0.007 | | 0.100 ± 0.017 | | 0.3909 | |  |


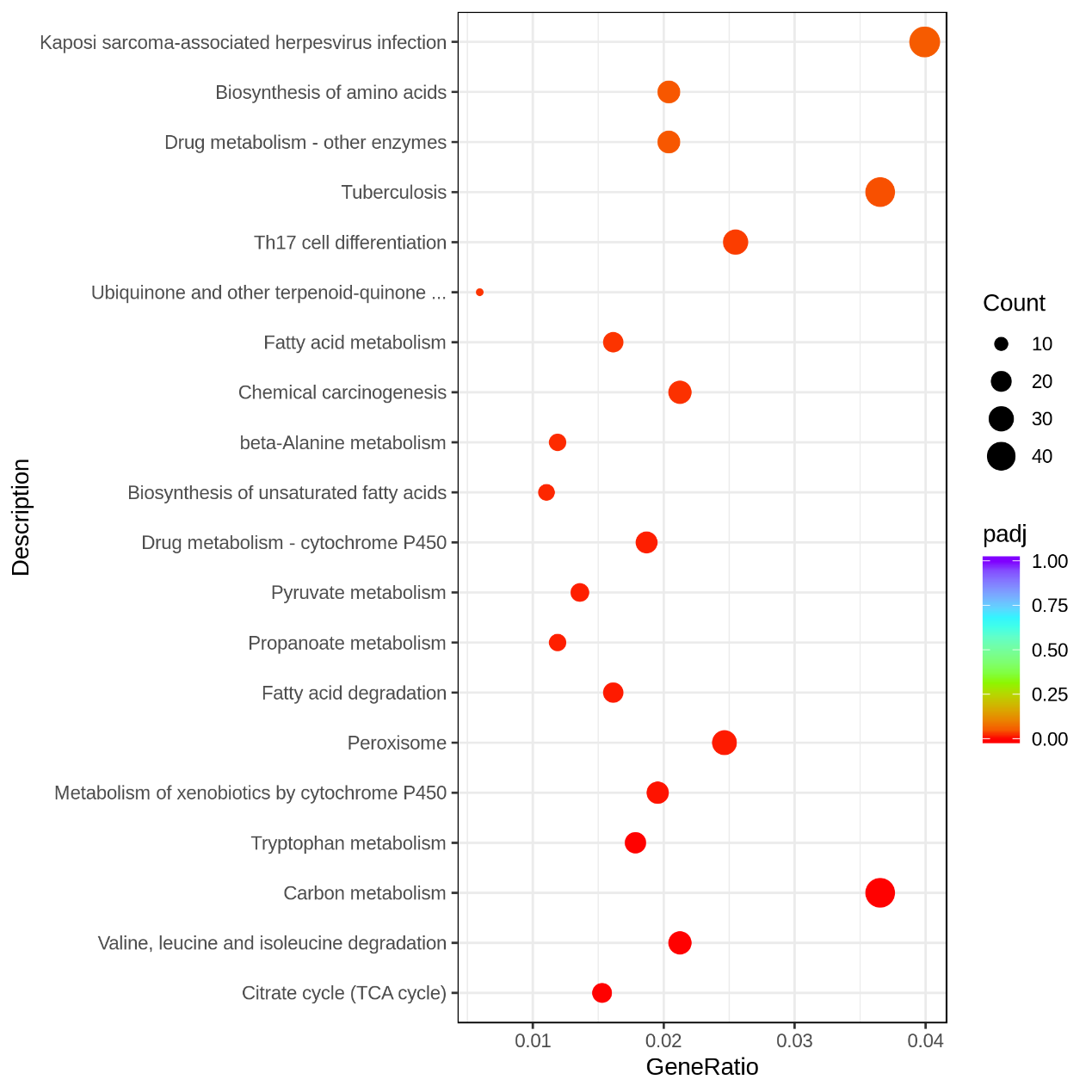


Fig. S1 Top 20 significant KEGG pathways in WAT of CAC and CONT mice.
